# Delayed Diagnosis of Cor Triatriatum Dexter: A Case Report and Comprehensive Review of Embryology, Imaging, and Management

**DOI:** 10.7759/cureus.93381

**Published:** 2025-09-27

**Authors:** Batoul Chaaban, Hassan Bitar, Ali Hamade, Imad Semaan, Abbas Rachid

**Affiliations:** 1 Cardiology, Lebanese University, Beirut, LBN; 2 Internal Medicine, Lebanese University, Beirut, LBN; 3 Internal Medicine, Saint Joseph University of Beirut, Beirut, LBN

**Keywords:** congenital cardiac anomalies, congenital cardiac surgery, cor triatriatum, cor triatriatum dexter, right-sided heart failure

## Abstract

Cor triatriatum dexter (CTD) is a rare congenital cardiac anomaly resulting from the persistence of the right valve of the sinus venosus, leading to partial or complete partitioning of the right atrium. It accounts for a minority of congenital heart defects and may present with various clinical features ranging from asymptomatic incidental findings to severe right-sided heart failure.

We report the case of a 45-year-old female with longstanding exertional dyspnea, peripheral edema, hepatomegaly, and severe bilateral varicose veins. Abdominal imaging revealed features of hepatic congestion, whereas echocardiography revealed a markedly dilated inferior vena cava (IVC) and a membrane at the junction of the IVC and right atrium. Transesophageal echocardiography confirmed a fibromuscular membrane consistent with CTD, partially obstructing systemic venous return. The patient also had a preserved left ventricular systolic function.

CTD arises from incomplete regression of the embryonic right venous valve. The degree of membrane perforation influences the severity of obstruction and clinical symptoms. While an incomplete CTD can be clinically silent, significant septation may lead to elevated right atrial pressures and systemic venous congestion. This case emphasizes the diagnostic utility of multimodality imaging in adults with unexplained right-sided heart failure symptoms and highlights CTD’s potential role in chronic venous disorders. This case adds to the limited literature on conservatively managed adult CTD, highlighting the impact of financial barriers on therapeutic decision-making.

CTD, although rare, should be considered in the differential diagnosis of right-sided congestion in adults, particularly when it is accompanied by unexplained IVC dilation, hepatic congestion, and chronic venous insufficiency. Early recognition through echocardiography and other imaging modalities is essential for guiding appropriate management and avoiding misdiagnosis.

## Introduction

Cor triatriatum is a rare congenital cardiac anomaly that accounts for approximately 0.1% of all congenital heart defects [1]. It is characterized by an abnormal membrane that separates either the left or right atrium, creating two distinct chambers and leading to variable degrees of inflow obstruction to the corresponding ventricle [2]. Normally, this valve regresses early in fetal life. In some cases, the anomalous membrane can mimic the echocardiographic appearance of Ebstein's anomaly, leading to diagnostic confusion [3]. When this anomaly occurs in the left atrium, it is referred to as the cor triatriatum sinister (CTS). In contrast, its right atrial counterpart is known as the cor triatriatum dexter (CTD). Although typically identified in infancy, CTD may occasionally be diagnosed in adulthood [3]. This condition can present in its isolated, classical form or in association with other congenital cardiac anomalies, ranging from simple to complex. Even within its classical form, multiple anatomical variants exist, often necessitating the use of multimodal imaging techniques for accurate characterization and optimal planning of percutaneous or surgical intervention [2]. An incomplete CTD membrane is often asymptomatic and considered an incidental finding because of its distinct embryologic origin from the atrial septum. Diagnostic identification of this membrane can be achieved via transthoracic echocardiography (TTE), transesophageal echocardiography (TEE), cardiac computed tomography angiography (CTA), or cardiac magnetic resonance imaging (MRI) [4]. We present a rare adult case of CTD, diagnosed after a presentation of chronic venous insufficiency and hepatic congestion.

## Case presentation

A 45-year-old female was referred to our center for further evaluation of disturbances in LFTs and hepatomegaly detected via abdominal ultrasound, which also revealed dilated hepatic veins, a dilated inferior vena cava (IVC), and features consistent with hepatic congestion. She reported a longstanding history of exertional shortness of breath, abdominal discomfort, and peripheral edema. Notably, she also described having severe bilateral varicose veins since a young age.

Upon examination, the patient was clinically stable, requiring no oxygen, but demonstrated reduced inspiratory effort. Physical examination revealed bilateral, prominent, purpuric, and dilated varicosities, associated with lower limb edema and erythema. A fourth heart sound (S4) was detected via cardiac auscultation.

**Figure 1:**
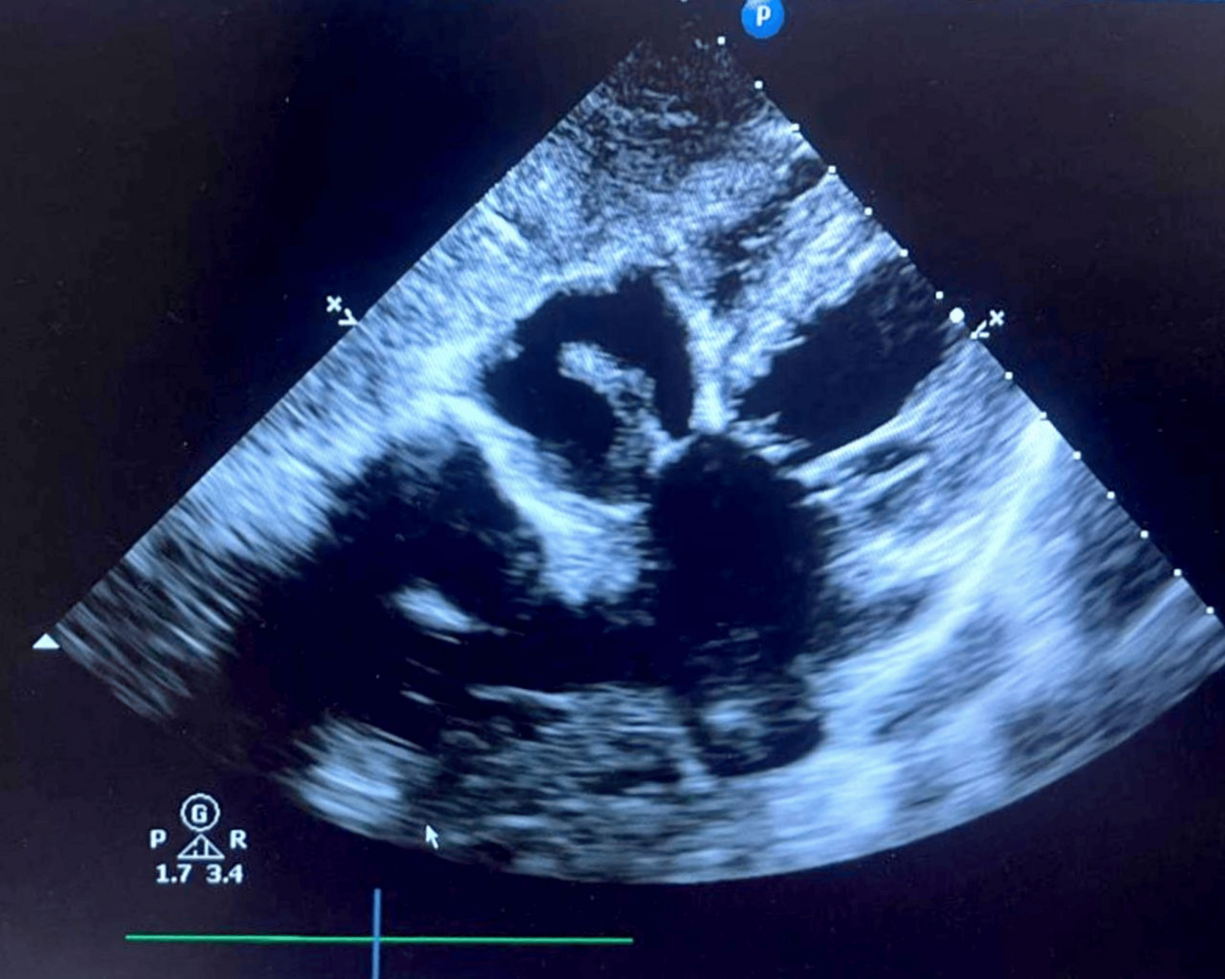
Sub-costal view of TTE showing prominent Eustachian valve in the middle of the right atrium TTE: transthoracic echocardiogram

A TTE was subsequently performed, which revealed a left ventricular ejection fraction of 54%, moderate mitral regurgitation, and mild pericardial effusion, with normal right ventricle size and function. The IVC was notably dilated (20 mm) and noncompressible, suggesting elevated right-sided filling pressures. The right atrium appeared normal in size; however, a membranous structure attached near the IVC orifice was visualized, raising suspicion of CTD. Based on these findings, a TEE was recommended for further evaluation (Figure 1).

**Figure 2:**
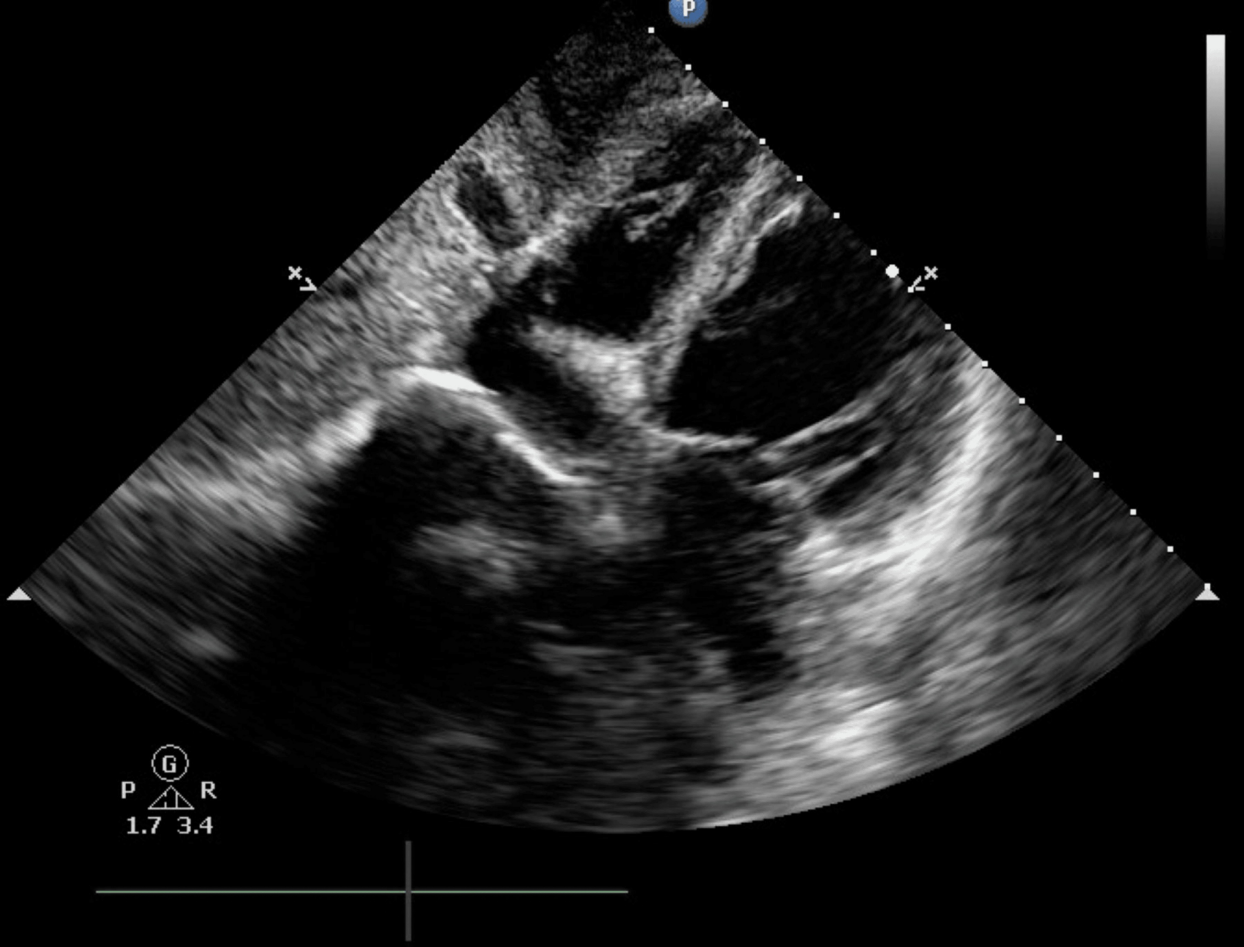
Subcostal TEE view revealing a prominent Eustachian valve within the right atrium TEE: transesophageal echocardiography

**Figure 3:**
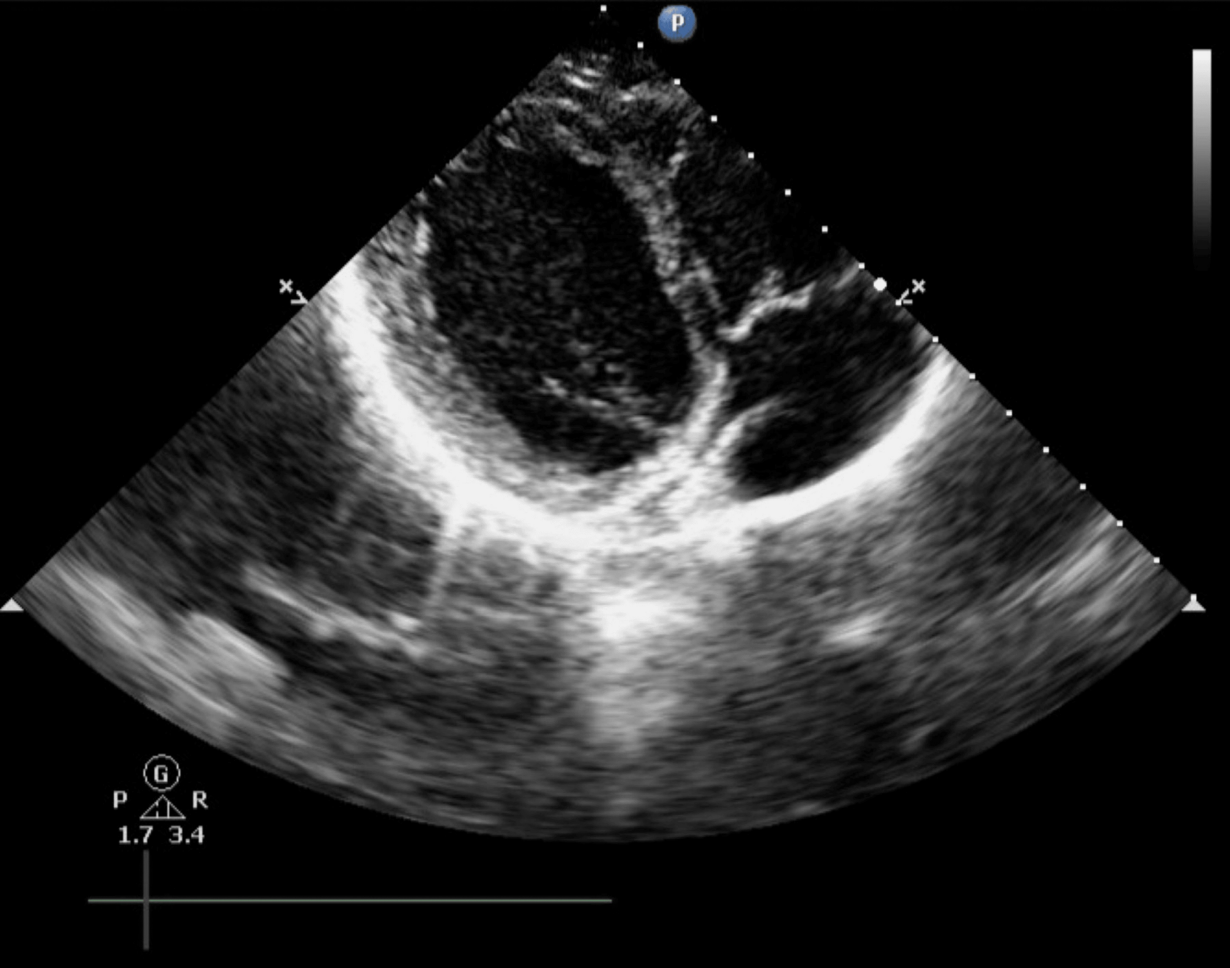
Subcostal TEE three chambers view revealing a prominent Eustachian valve within the right atrium TEE: transesophageal echocardiography

**Figure 4:**
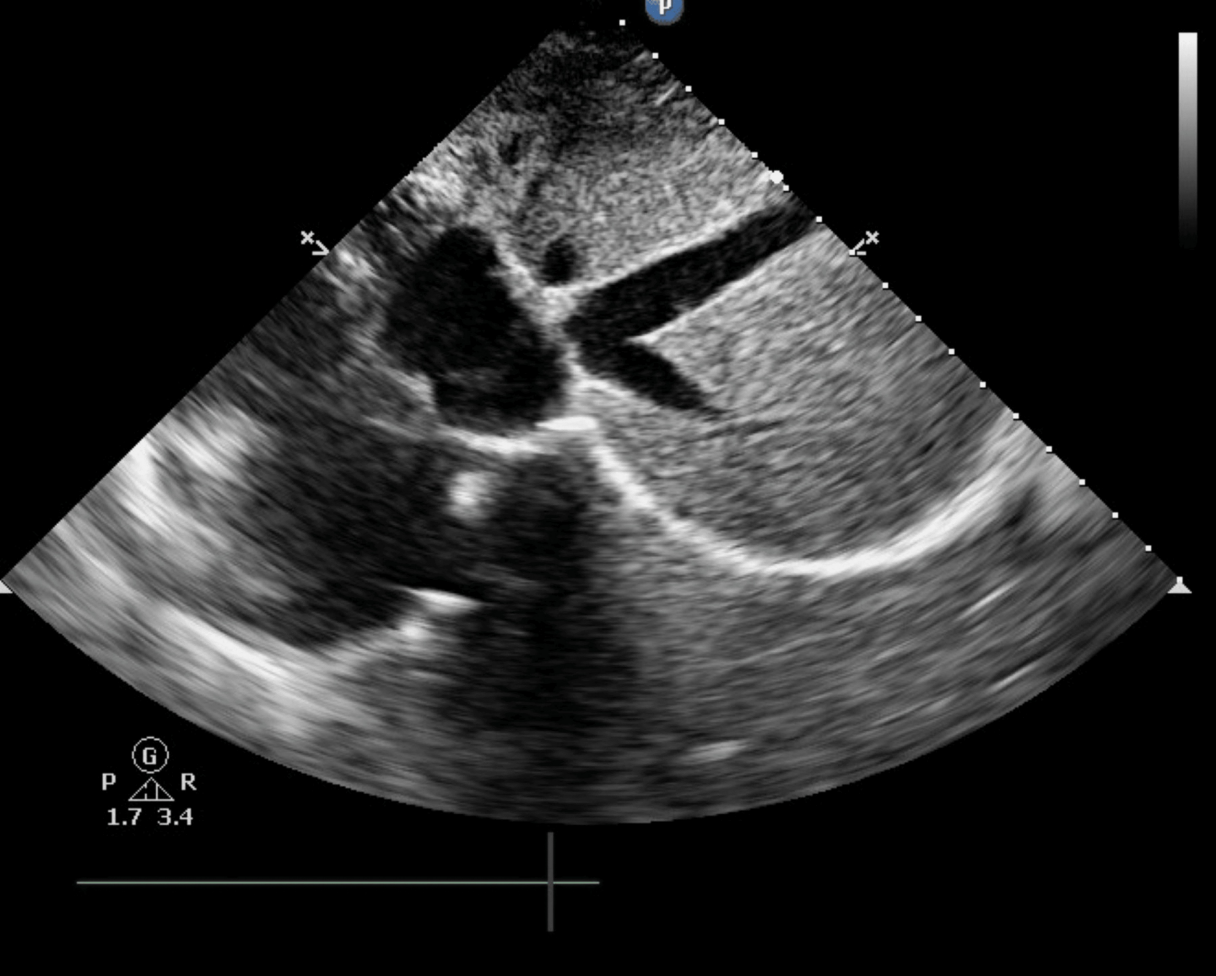
Subcostal TEE view showing the Eustachian valve obstructing the IVC TEE: transesophageal echocardiography, IVC: inferior vena cava

TEE confirmed the presence of a membrane at the junction of the IVC and right atrium (Figure 2), which was consistent with a prominent Eustachian valve remnant (Figure 3). This membrane was found to partially obstruct the IVC inflow, with persistent dilation of the IVC, supporting the diagnosis of CTD (Figure 4). The patient was managed conservatively, with optimization of medical therapy aimed at controlling symptoms and reducing venous congestion. The patient remained stable and did not require any invasive intervention. This case underscores how socioeconomic factors may shape the management decisions in patients with anatomically significant but clinically compensated CTD.

## Discussion

CTD: embryological basis and clinical relevance

CTD is a rare congenital anomaly of the right atrium that results from the persistence of the right leaflet of the venous sinus valve, which normally regresses around the 12th week of gestation. During embryonic development, the common atrium is separated from the venous sinus by a valve with two leaflets. The left leaflet becomes incorporated into the interatrial septum, contributing to the septum secundum, while the right leaflet is normally reabsorbed, leaving behind two structures: the Eustachian valve at the junction of the IVC and the coronary valve at the orifice of the coronary sinus. In CTD, however, persistence of this embryonic valve leads to a fibromuscular membrane that partitions the right atrium into two chambers: a posterior (sinus venarum) chamber that receives systemic venous return from the superior vena cava and IVC, and an anterior chamber that communicates with the tricuspid valve and right ventricle [3].

The clinical manifestations of CTD depend on the degree and morphology of this atrial septation. In cases of incomplete or fenestrated septation, blood flow between the two right atrial chambers remains relatively unimpeded, and the condition may remain asymptomatic or be discovered incidentally on imaging or autopsy. However, in cases of complete or nearly complete septation, the obstructive membrane may impede venous return or tricuspid inflow, leading to elevated right atrial pressures, systemic venous congestion, cyanosis, or even right-sided heart failure in neonates and infants. Moreover, CTD is frequently associated with other congenital cardiac anomalies, including atrial septal defects (ASDs) and Ebstein anomaly of the tricuspid valve, which may further influence clinical presentation and hemodynamics [3].

Morphologic associations in hypoplastic right heart syndrome

Anatomopathological studies have explored the relationship between CTD and other structural abnormalities of the right heart, particularly in the context of hypoplastic right heart syndrome. In a study involving 20 hearts with right heart underdevelopment and 17 structurally normal control hearts, enlarged Eustachian valves were identified in 80% (n = 16) of the hypoplastic group, with one case (5%) showing mild enlargement and three (15%) showing hypoplastic valves. Notably, CTD was identified in 25% (n = 5) of these hearts. In contrast, among the control samples, a prominent Eustachian valve was observed in only one sample (6%), whereas it was normal in five (29%) and nearly absent in eight (47%) samples. These findings suggest that enlargement of the Eustachian valve and persistence of the right valve of the sinus venosus, both of which are embryologic remnants, may contribute significantly to aberrant right atrial anatomy. Furthermore, in fetal and neonatal echocardiographic studies, a prominent Eustachian valve directing systemic venous blood across an ASD has been documented in up to 80% of cases, with CTD coexisting in approximately 25% of cases [5]. These data reinforce the relevance of CTD in the broader spectrum of right-sided congenital heart disease and highlight its potential role in fetal circulation anomalies and postnatal hemodynamic compromise.

Associated cardiac anomalies in CTS and CTD

Cor triatriatum, particularly CTD, is frequently associated with a wide spectrum of congenital cardiac abnormalities. While it may present as an isolated anomaly, it often coexists with other structural defects, particularly those affecting the right side of the heart. A high incidence of right-sided congenital anomalies has been reported in association with CTD. These include pulmonary artery stenosis or atresia, tricuspid valve abnormalities, ASDs, and Ebstein’s anomaly [6]. CTD may present with features similar to Ebstein anomaly, complicating the clinical diagnosis [7].

Several case reports highlight the diversity of associated defects. For example, Alvarez-Santana et al. described a case of CTD associated with Ebstein anomaly, tricuspid valve lesions, and an ASD [8]. Alghamdi also noted that CTD can occur as an isolated lesion or in combination with other abnormalities [9]. Furthermore, Özmen et al. documented a rare triad of CTD, a mitral valve blood cyst causing regurgitation, and an atrial septal aneurysm, underscoring the broad anatomical spectrum that may be involved [10].

Pulmonary hypertension is another important comorbidity. Meher et al. reported a case of neonatal CTD complicated by pulmonary hypertension, emphasizing the hemodynamic burden that can result from venous inflow obstruction [11]. Additionally, in the long-term experience at the Mayo Clinic, the most frequently encountered associated anomalies with cor triatriatum were ASDs, patent foramen ovale, and pulmonary valve defects [12].

While CTD is more commonly associated with right-sided lesions, left-sided abnormalities may also occur. For instance, Özmen et al. described a mitral valve blood cyst in conjunction with CTD [13], and another case report identified mitral regurgitation as part of the clinical presentation [14]. These observations align with emerging data suggesting that CTD, even when isolated, may coexist with a broader range of valvular defects, including aortic and mitral valve disease.

Rare associations, such as the levoatrial cardinal vein, have also been described in patients with cor triatriatum, particularly CTS. This venous anomaly appears more frequently in patients with cor triatriatum than in other congenital heart diseases and may contribute to complex hemodynamic presentations [15]. A broader analysis reveals that cor triatriatum, particularly CTS, is often associated with secundum ASDs, patent foramen ovale, tricuspid regurgitation, partial anomalous pulmonary venous return, myxomatous mitral valve disease with regurgitation, and pulmonary vein stenosis [12]. These associations highlight the importance of detailed imaging and clinical suspicion in atypical presentations of the right-sided heart.

Diagnostic classification and imaging modalities in CTD

Accurate diagnosis, risk stratification, and optimal surgical or interventional planning in CTD require a thorough understanding of its anatomical variations and associated cardiac anomalies. Multimodality imaging, particularly TTE, TEE, cardiac MRI, and CT, is essential to fully delineate the anatomical context, especially in patients being considered for surgical or catheter-based interventions [16].

Due to its rarity, with fewer than 100 cases reported in the literature, no standardized classification system for CTD currently exists. The clinical manifestations of CTD are highly variable and depend largely on the degree of right atrial septation caused by the persistent fibromuscular membrane. The presence and size of membrane perforations, the occurrence of right-to-left shunting, and the coexistence of other congenital anomalies further influence symptomatology. In cases where septation is partial or the membrane is fenestrated, the condition may remain asymptomatic or present with mild cyanosis. More extensive septation may result in significant obstruction to venous inflow, leading to elevated right atrial pressures, systemic venous congestion, and, in some cases, right-sided heart failure. Cyanosis has also been documented in the context of severe right inflow obstruction [16].

Historically, CTD was often identified post-mortem due to its subtle clinical signs and the limited diagnostic capabilities available in earlier eras [17]. However, with the advancement of cardiovascular imaging, particularly non-invasive modalities, diagnosis in living patients has become increasingly feasible and reliable. Since the 1990s, echocardiography has emerged as the cornerstone of CTD diagnosis. Two-dimensional TTE enables the direct visualization of intra-atrial membranes, while color Doppler imaging reveals the associated turbulent flow patterns. Continuous-wave Doppler further aids in assessing hemodynamic impact by quantifying pressure gradients across the membrane [18,19].

In a 22-year retrospective study from the Royal Children’s Hospital in Melbourne, Australia, involving 28 cases of cor triatriatum, TTE successfully established the diagnosis preoperatively in 96% of patients. Notably, in 32% of cases with atypical anatomy, confirmatory cardiac catheterization was required. This underscores both the high diagnostic accuracy of echocardiography and the potential need for supplemental imaging in complex presentations [20].

TTE remains the first-line diagnostic modality in most reported cases of CTD [21-27], while TEE is often employed to confirm the diagnosis and provide enhanced anatomical detail when TTE findings are inconclusive [28-30]. More recently, cardiac MRI has gained traction due to its high spatial resolution, allowing for detailed characterization of the membranous structure and comprehensive assessment of right ventricular volumes and function. In a comparative study assessing pulmonary venous anomalies, which included cases of cor triatriatum, MRI demonstrated a diagnostic sensitivity of 95%, outperforming both cardiac angiography (69%) and echocardiography (38%) [31]. Cardiac CT is also increasingly recognized as a valuable non-invasive tool, particularly for visualizing cardiac and vascular structures in complex congenital anomalies, including CTD and coexisting conditions such as coronary artery disease [32]. Together, these imaging techniques provide a robust framework for accurate diagnosis and guide tailored therapeutic decision-making in patients with CTD.

Therapeutic approaches in cor triatriatum: surgical, conservative, and emerging interventions

Surgical intervention remains the definitive treatment for symptomatic CTS, especially when obstruction leads to elevated transmembrane pressure gradients (typically ≥8 mm Hg) or when significant associated congenital cardiac lesions are present [33]. The earliest successful surgical repair was performed in 1955 by Lewis et al. using cardiopulmonary bypass, marking a pivotal advancement in the treatment of congenital heart disease [34].

Since then, surgical membrane resection under cardiopulmonary bypass has become the standard of care, particularly when other cardiac anomalies such as ASD or valvular abnormalities coexist. In a large surgical series from the Mayo Clinic involving 25 patients operated on over five decades, favorable outcomes were consistently reported across all age groups, with additional procedures such as ASD repair and valve surgery commonly performed [12]. A five-year survival rate of 100% in patients with biventricular physiology further highlights the efficacy of early surgical management. However, not all patients are suitable candidates for surgery [35].

Our case is a prime example of this approach. A 45-year-old woman presented with chronic hepatic congestion, elevated liver function tests, bilateral varicosities, and a longstanding history of exertional dyspnea and peripheral edema. TTE and TEE revealed a prominent membranous remnant at the junction of the IVC and right atrium, consistent with CTD. Despite partial IVC inflow obstruction and IVC dilation, the patient remained hemodynamically stable, with no overt signs of right heart failure requiring urgent surgical correction. Unfortunately, due to financial limitations, surgical intervention was not feasible. Therefore, a conservative strategy with close clinical and imaging follow-up was adopted. Our case uniquely highlights how socioeconomic constraints can influence the management of anatomically significant, yet clinically compensated, CTD.

Although surgical resection remains the gold standard, evolving transcatheter approaches have demonstrated promise. Balloon dilatation, either alone or in conjunction with ASD occluder placement, has been reported to yield favorable outcomes, particularly in adult patients with isolated membrane-related obstruction [36,37]. Nonetheless, the limited availability and experience with these minimally invasive strategies, especially in pediatric or resource-limited settings, reinforces the role of surgery as the preferred approach when accessible [36].

## Conclusions

CTD is a rare but clinically meaningful congenital cardiac anomaly that may remain undiagnosed until adulthood, particularly when membrane septation is incomplete and symptoms are subtle. This case demonstrates how CTD can manifest as chronic systemic venous hypertension, evidenced by hepatic congestion and peripheral venous insufficiency due to partial IVC inflow obstruction. Accurate diagnosis requires a high index of suspicion and multimodal imaging, with TEE being particularly valuable. Although surgical resection remains the definitive treatment for symptomatic or obstructive CTD, conservative management is a reasonable alternative in stable patients or those with limited access to surgery. Ultimately, a tailored approach that incorporates clinical presentation, imaging severity, and socioeconomic context is essential for optimizing patient outcomes. Routine echocardiographic follow-up remains crucial to detect disease progression or emerging complications.
